# Pigment Epithelial-Derived Factor in Pancreatic and Liver Cancers—From Inflammation to Cancer

**DOI:** 10.3390/biomedicines12102260

**Published:** 2024-10-04

**Authors:** Sara Pączek, Monika Zajkowska, Barbara Mroczko

**Affiliations:** 1 Department of Biochemical Diagnostics, University Hospital in Białystok, 15-269 Białystok, Poland; sara.paczek@uskwb.pl (S.P.); barbara.mroczko@umb.edu.pl (B.M.); 2 Department of Neurodegeneration Diagnostics, Medical University of Białystok, 15 A, Waszyngtona St., 15-269 Białystok, Poland; 3 Department of Biochemical Diagnostics, Medical University of Białystok, 15-089 Białystok, Poland

**Keywords:** PEDF, cancer, gastrointestinal malignancies, pancreatic cancer, hepatocellular carcinoma, biomarkers

## Abstract

Gastrointestinal (GI) cancers are among the leading causes of mortality worldwide. Despite the emergence of new possibilities that offer hope regarding the successful treatment of these cancers, they still represent a significant global health burden. These cancers can arise from various cell types within the gastrointestinal tract and may exhibit different characteristics, behaviors, and treatment approaches. Both the prognosis and the outcomes of GI treatment remain problematic because these tumors are primarily diagnosed in advanced clinical stages. Current biomarkers exhibit limited sensitivity and specificity. Therefore, when developing strategies for the diagnosis and treatment of GI cancers, it is of fundamental importance to discover new biomarkers capable of addressing the challenges of early-stage diagnosis and the presence of lymph node metastases. Pigment epithelial-derived factor (PEDF) has garnered interest due to its inhibitory effects on the migration and proliferation of cancer cells. This protein has been suggested to be involved in various inflammation-related diseases, including cancer, through various mechanisms. It was also observed that reducing the level of PEDF is sufficient to trigger an inflammatory response. This suggests that PEDF is an endogenous anti-inflammatory factor. Overall, PEDF is a versatile protein with diverse biological functions that span across different tissues and organ systems. Its multifaceted activities make it an intriguing target for therapeutic interventions in various diseases, including cancer, neurodegeneration, and metabolic disorders. This review, for the first time, summarizes the role of PEDF in the pathogenesis of selected GI cancers and its potential utility in early diagnosis, prognosis, and therapeutic strategies for this malignancy.

## 1. Introduction

### 1.1. Pigment Epithelial-Derived Factor

**Figure 1:**
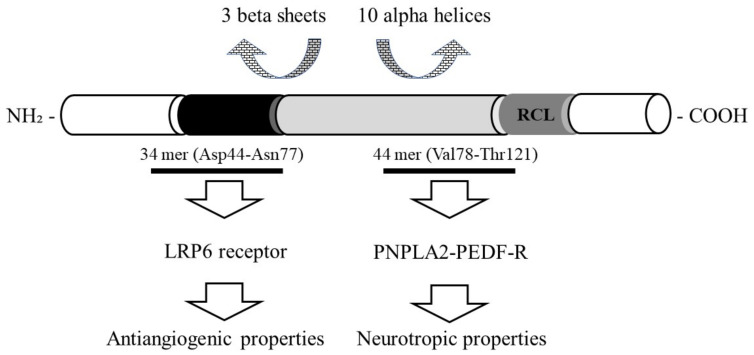
The schematic structure of PEDF consists of several distinct domains and regions, each of which is responsible for specific biological activities, such as antiangiogenic, neurotrophic, and anti-tumor effects. Abbreviations: LRP6—LDL Receptor-Related Protein 6, PNPLA2—photoreceptor cells express the patatin-like phospholipase domain-containing 2, RCL—reactive center loop.

Pigment epithelial-derived factor (PEDF) is a glycoprotein encoded by the SER-PINF1 gene and has a molecular weight of 50 kDa. It belongs to the noninhibitor serine protease inhibitor (Serpin) family [1]. Similar to other serpins, PEDF has a tertiary structure consisting of three beta sheets and ten alpha helices. In addition, it contains a typical reactive center loop (RCL) in its structure, which is located near the C-terminus [2,3] (Figure 1).

**Figure 2:**
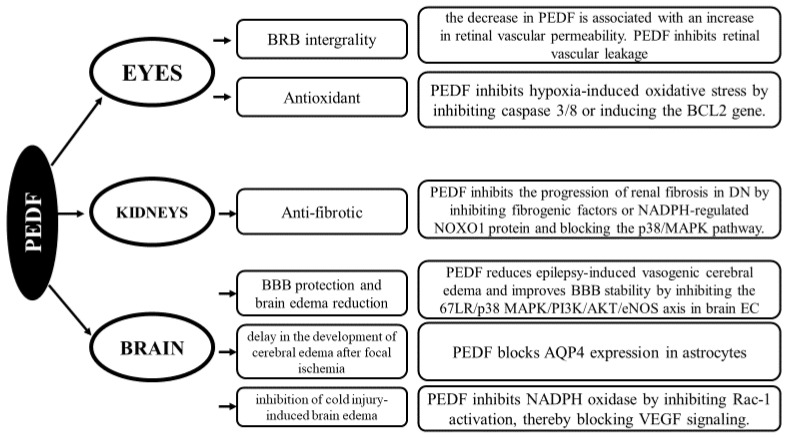
PEDF plays an important role in microcirculation, which refers to the circulation of blood in the smallest blood vessels, including arterioles, capillaries, and venules. PEDF primarily exerts its effects through antiangiogenic, anti-inflammatory, and antioxidant actions, which influence the behavior of endothelial cells, vascular permeability, and overall vascular health in the microcirculatory system. Abbreviations: 67LR—67 kDa laminin receptor, AKT—protein kinase B, AQP4—aquaporin-4, BBB—blood–brain barrier, BCL2—B-cell lymphoma 2, BRB—blood–retina barrier, DN—diabetic nephropathy, EC—endothelial cells, eNOS—endothelial nitric oxide synthase, MAPK—mitogen-activated protein kinase, NADPH—nicotinamide adenine dinucleotide phosphate hydrogen, NOXO1—NADPH oxidase organizer 1, PEDF—pigment epithelium-derived factor, PI3K—phosphoinositide 3-kinases, VEGF—vascular endothelial growth factor [15,16,21,22,23,24].

However, unlike standard serpins that inhibit serine proteases, the reactive center loop (RCL) region of PEDF contains specific amino acid substitutions, which consequently lead to this protein’s inability to bind to target cysteine/serine proteases [4]. PEDF interacts with various but specific types of receptors, including pigment epithelium-derived factor receptor (PEDFR) or low-density lipoprotein receptor-related protein (LRP6), which guarantees that the biological activity of this protein is characterized by extensive pleiotropism [5]. Although the physiological concentration of PEDF in the serum is still a subject of controversy due to the heterogeneous values obtained depending on the method used, it has been shown that the level of this protein decreases with age [6]. PEDF functions by inhibiting the proliferation and migration of endothelial cells and by activating the apoptosis pathway in these cells [6,7]. It has been shown that the PEDF protein plays a pivotal role, initially in organogenesis and subsequently in maintaining the homeostasis of mature human organs [8]. Both structural defects and deficiencies in the expression of this molecule are closely related to the progression of angiogenic diseases [8,9]. It was also shown that the lack of PEDF in Serpinf1 null × rd10 mice resulted in an increased susceptibility to retinal degeneration compared to that of rd10 mice [10]. PEDF has been found to play a role in several biological processes, such as neurogenesis, neuroprotection, antiangiogenesis, retinal protection, stem cell renewal, and inflammation, making it a vital protein in the body [11,12]. It also inhibits retinal neovascularization and endothelial cell proliferation. As we know, PEDF can be localized either in the nucleus or in the cytosol [1] and can also be secreted—for example by human fetal retinal pigment epithelial cells [13]. This evidence suggests that the functions of this protein can be performed inside the cell as well as outside. The extracellular localization of PEDF is associated with its presence in body fluids, where it reaches high concentrations, as well as in the extracellular matrix, where this protein interacts with glycosaminoglycans and collagen [14]. Under physiological conditions, PEDF’s role has also been attributed to maintaining the balance of microcirculation in organs such as the brain, heart, kidneys, and the retina of the eye [15,16,17,18]. Additionally, the importance of this protein in organ development and pathophysiology is influenced by its expression levels. For instance, PEDF deficiencies in the kidneys and the eye significantly impact the density of the capillary plexus, both in physiological and pathological conditions [19]. Furthermore, PEDF levels have been shown to be closely related to the development of coronary artery disease as well as diabetic microvascular disease [20]. The schematic involvement of PEDF in microcirculatory diseases is presented in Figure 2.

### 1.2. Gastrointestinal Malignancies

Many epidemiological studies suggest that inflammation is one of the main factors leading to or promoting cancer development including gastrointestinal (GI) cancers. Pancreatic cancer (PC) is a prominent contributor to global cancer mortality. This cancer is marked by rapid progression, unfavorable prognosis, and limited treatment options [25]. The regions with the highest incidence of PC are North America, Europe, as well as Australia [26]. The precise etiology of PC development remains unclear. Nevertheless, various factors have been proposed as contributors to an elevated risk of this malignancy. These factors have been typically categorized into two groups: modifiable and non-modifiable [27]. The former includes obesity, cigarette smoking, and heightened consumption of animal fat, while the non-modifiable risk factors encompass sex, age, inherited genetic predisposition, and chronic pancreatitis (CP) [28]. A primary challenge in diagnosing PC lies in the fact that the majority of cases remain asymptomatic during the early stages of the disease. Symptoms such as abdominal pain, jaundice, or unintended weight loss should not be overlooked [29]. The majority of pancreatic tumors typically localize in the head of the pancreas, causing symptoms like biliary duct obstructions. In contrast, tumors situated in the body or tail of the pancreas tend to remain asymptomatic until the disease progresses to a more advanced stage [30]. The diagnosis of PC involves a comprehensive process with multiple steps. Presently, clinical practice relies on imaging techniques like transabdominal ultrasonography (USG), computed tomography (CT), as well as magnetic resonance imaging (MRI) [31]. More specific methods include MR cholangiopancreatography (MRCP) and endoscopic ultrasound (EUS) [32]. Despite their utility, these diagnostic tools are constrained by elevated costs and the use of invasive procedures, limiting their overall effectiveness. The recognized tumor markers for monitoring pancreatic cancer (PC) patients include measuring serum concentrations of carcinoembryonic antigen (CEA) or carbohydrate antigen 19-9 (CA 19-9) [33]. However, despite their established role in follow-up, these biomarkers represent low specificity and sensitivity during the early stages of the disease, rendering them impractical for screening [34]. Hence, there is a pressing demand to discover novel compounds that can aid in the early diagnosis of individuals with this form of cancer.

Liver cancer (LC) ranks as the third leading cause of cancer-related deaths glob-ally. Hepatocellular carcinoma (HCC) constitutes the most prevalent histological type of liver cancer, accounting for over 80% of cases [35]. The global distribution of liver cancer varies significantly, with the highest burden in regions with endemic hepatitis B virus (HBV) infection, notably in Asian countries, particularly in East and Southeast Asia [36]. Recognized risk factors for LC are chronic infection with the HBV or hepatitis C virus (HCV), excessive alcohol consumption as well as non-alcoholic fatty liver disease (NAFLD) [37]. Symptoms are nonspecific and may involve fatigue and loss of appetite. Regarding liver cancer (LC), which shares similarities with pancreatic cancer (PC), there can be weight loss and jaundice due to compromised liver function. Furthermore, pain may be experienced in the upper right side of the abdomen [38]. Diagnosis involves a comprehensive approach, combining medical history and physical examination with various tests. Imaging techniques like ultrasound, CT, and MRI are commonly used, along with blood tests that may include the classical tumor marker—alpha-fetoprotein (AFP) [39].

It is important to note that despite advancements in diagnostic methods for both PC and LC, a biopsy remains essential to definitively confirm the diagnosis.

Advances in the treatment of this group of cancers are closely linked to the global development in diagnostics. Over the past few years, oncological treatment has made significant strides, which are largely attributable to the expansion of the options for combined procedures. Nevertheless, it is evident that surgical intervention remains the predominant treatment modality for most cancer types [40,41]. However, the effectiveness of treatment and prognosis depends on numerous factors, including those that depend on the patient, but also to a large extent on the close cooperation of doctors. This underscores the importance of an interdisciplinary approach to patient management. Additionally, progress can be achieved through the development of molecularly targeted therapies, which significantly enhance the chances of a cure [42,43,44].

## 2. PEDF in Tumor

Numerous studies exploring cancer biology have identified several fundamental steps responsible for cancer progression [45]. The primary contributors are tumor growth, invasion, and metastasis. Each of these stages is significantly influenced by the interaction between cancerous cells and the surrounding vascular environment [46,47]. The phenomenon of angiogenesis has garnered considerable attention from scientists, as it plays a vital role in tumor survival by providing both oxygen and nutrients [48,49]. The process of angiogenesis begins with the disturbance of homeostasis between pro-angiogenic factors, such as VEGF, and antiangiogenic factors, such as PEDF [50]. In neoplastic tumors, angiogenesis significantly differs from natural, physiological vasculogenesis. This distinction is believed to be attributed to chronic inflammation and the pronounced hypoxia that develops within cancerous tissues [51].

There has been controversy and debate in the scientific community regarding the pathophysiological role of PEDF in various diseases, including cancer. While PEDF is traditionally known as an antiangiogenic and tumor-suppressive protein, there have been reports suggesting it may also act as tumor progressor in certain contexts [52].

The overexpression of PEDF in cancer can have complex implications, as it often functions as a tumor suppressor but can exhibit paradoxical effects depending on the context and the cancer type. Overexpression of PEDF in MSCs has been observed to effectively decrease angiogenesis by countering VEGF signaling, thereby restricting the tumor’s blood supply. This reduces the ability of gliomas to grow and spread. The importance of PEDF overexpression lies in its ability to inhibit glioma angiogenesis and reduce tumor growth, making it a potential therapeutic tool for glioma treatment [53]. This research highlights the dual role of PEDF in both suppressing blood vessel formation and directly impeding tumor proliferation, particularly when it is delivered via genetically modified mesenchymal stem cells.

**Figure 3:**
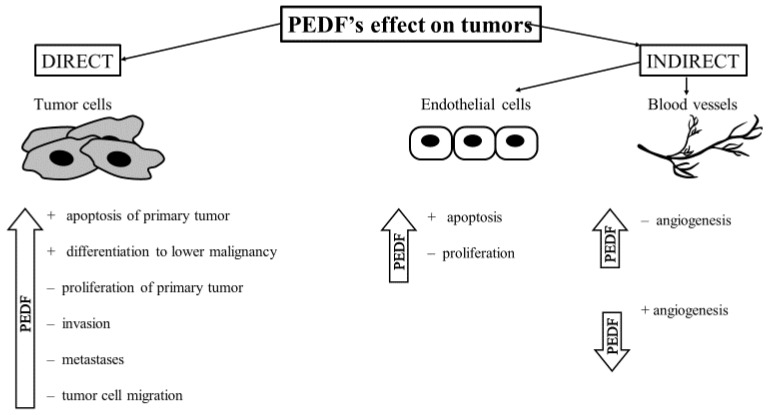
The effect of PEDF on tumors. These effects contribute to its overall anti-tumor activity by targeting both the tumor cells and the surrounding microenvironment, particularly through the regulation of angiogenesis, apoptosis, and immune responses. Abbreviations: PEDF—pigment epithelium-derived factor.

The anticancer effect is one of PEDF’s most intriguing properties. This protein is able to affect tumor regression both directly—by impacting cancer cells directly—or by indirectly affecting endothelial cells and blood vessels [54], as shown in Figure 3.

The biological processes inhibited by PEDF activity primarily include angiogenesis, proliferation and invasion, migration of tumor cells, as well as the formation of distant metastases. While the precise mechanism through which PEDF impedes angiogenesis remains incompletely elucidated, there is a proposition that it is associated with an augmentation in gamma-secretase activity [55,56]. Gamma-secretase is a multi-subunit protease complex, which is involved in the proteolytic cleavage of transmembrane proteins, including Notch and other receptors like VEGFR-2, which is a critical step for signaling through the receptor. The cleavage of that receptor prevents the full activation of VEGF signaling, which means that even if VEGF is present and attempts to bind to the VEGFR-2, the receptor is either degraded or its signaling potential is reduced [57]. PEDF can inhibit gamma-secretase activity, thereby preventing the cleavage of VEGFR-2. This interference blocks the formation of the intracellular domain fragment of VEGFR-2, which is necessary for full receptor activation and downstream signaling. By inhibiting gamma-secretase activity and preventing VEGFR-2 cleavage, PEDF effectively reduces the intracellular signaling that VEGF would typically activate [58]. This inhibition disrupts the angiogenic signaling cascades (e.g., PI3K/Akt, MAPK/ERK), leading to reduced endothelial cell proliferation, migration, and survival [59]. This ultimately results in decreased angiogenesis. In addition, PEDF can also compete with VEGF by binding to its receptors or inducing apoptosis in endothelial cells, further contributing to the inhibition of VEGF signaling and angiogenesis [60]. Moreover, PEDF, which is the main antagonist of vascular endothelial growth factor (VEGF), can regulate its proteolysis, and thus successfully inhibits vascular permeability and VEGF-driven angiogenesis. Therefore, the above-mentioned crosstalk between PEDF and VEGF signaling serves as critical checkpoint in the regulation of angiogenesis. The ability of cancer cells to both proliferate and invade healthy tissue is one of the key characteristics of cancer [61,62,63]. It has been shown that PEDF is able to inhibit the proliferation of endothelial cells, and when PEDF is exogenously supplied to culture endometrial cancer cells, it leads to a reduction in their proliferation. The exact relationship that explains PEDF’s pathway to limiting cell invasion is not fully understood, but matrix metalloproteinases’ (MMPs) involvement is suggested as one of the potential mechanisms. MMPs’ ability to degrade the extracellular matrix prevents cancer cells from breaking through tissue barriers and spreading to distant metastases [64]. However, the presence of metastases is one of the most important factors associated with the mortality of cancer patients, regardless of the type [65]. They occur when cancer cells separate from the primary tumor and then travel to the blood vessels. At a later stage, these cells “travel” to distant parts of the body and attach to the endothelium of isolated organs, creating completely new tumor colonies [66]. The role of PEDF in the inhibition of metastases comes down to the previously mentioned ability to switch off MMPs, as well as the VEGF/PEDF ratio, which is a kind of angiogenic switch in melanoma metastases [67,68].

**Figure 4:**
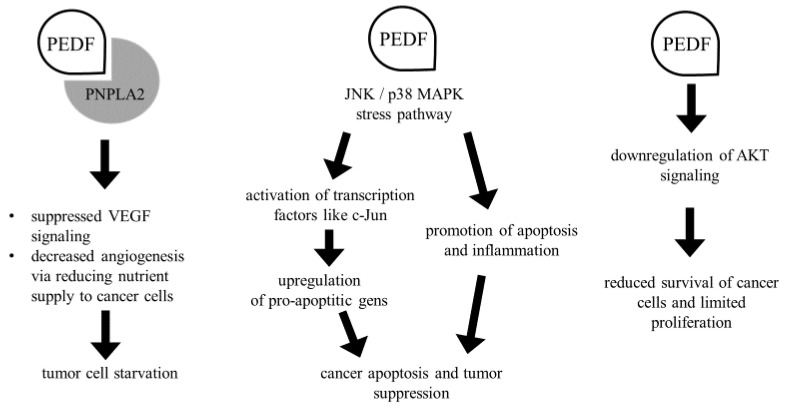
The influence of PEDF through VEGF, JNK, p38/MAPK and AKT signaling in cancer. Abbreviations: AKT—Protein Kinase B, c-Jun—N-terminal Kinase, JNK—c-Jun N-terminal Kinase, MAPK—mitogen-activated protein kinase, PEDF—Pigment Epithelium-Derived Factor, PNPLA2—patatin-like phospholipase domain containing 2 (also known as adipose triglyceride lipase, ATGL), VEGF—vascular endothelial growth factor.

The second category of biological anticancer processes influenced by PEDF involves the stimulation of primary tumor apoptosis and differentiation towards a less malignancy [69]. When assessing the anticancer effect of PEDF, the regulation of apoptosis was shown as its key biological benefit [70]. One of the fundamental mechanisms affecting PEDF antiangiogenesis is its tendency to selectively stimulate endothelial apoptosis. PEDF stimulates apoptosis through many pathways, the most famous of which are the extrinsic pathway, which involves the death receptor located on the cell surface, and the intrinsic pathway, also called the mitochondrial pathway due to the final increase in mitochondrial permeability [71]. The expression of the pro-apoptotic FAS receptor in resting endothelial cells is usually low but increases with the presence of angiogenic factors such as VEGF. As a consequence, stimulated cells become sensitive to apoptosis. In cancer, PEDF can directly induce apoptosis in cancer cells by upregulating pro-apoptotic factors, primarily through the FAS/FASL pathway, while also downregulating antiapoptotic proteins like the B-cell lymphoma 2 (BCL2) family of proteins, which are associated with the intrinsic pathway [72]. Additionally, it has been suggested that PEDF mediates endothelial cell apoptosis by activating c-Jun NH2-terminal kinase (JNK), leading to the inhibition of cellular FLICE-like inhibitor proteins (c-FLIP), thereby inducing a pro-apoptotic state [62]. This protein is an intracellular inhibitor of caspase-8 activation, effectively preventing death signaling mediated by all commonly known death receptors, including, but not limited to, FAS, tumor necrosis factor receptor (TNF-R), and TNF-related apoptosis-inducing ligand receptors (TRAIL-R). Furthermore, PEDF, in addition to its role in inhibiting angiogenesis, has been shown to induce neuroendocrine differentiation of prostate cancer cells [73,74]. It is suggested that PEDF’s ability to inhibit cell growth as well as differentiate cancer cells into a less malignant phenotype requires a very complex system to regulate these processes [75,76]. Figure 4 presents the PEDF signaling pathways that have the most involvement in cancer.

Despite its tumor-suppressing actions, PEDF may act as a tumor progressor. This role is influenced by various factors, including the tumor type or microenvironment. In some cancers chronic inflammation is linked to cancer progression, because it can promote cellular proliferation, enhance signals and stimulate angiogenesis. In most settings, PEDF is responsible for angiogenesis inhibition [77]. However, in hypoxic environments, which are common for solid tumors, the balance between pro-angiogenic and antiangiogenic factors shifts. Under numerous low-oxygen conditions, PEDF expression can be altered, leading to changes in its effects on endothelial cells. In this context, PEDF may not be as effective at counteracting VEGF, potentially allowing angiogenesis to proceed unchecked [78].

The interaction of PEDF and laminin receptor (LR) or adipose triglyceride lipase (ATGL) has also been shown to mediate its biological effects. The expression of these receptors or their downstream signaling pathways may be altered, potentially transforming PEDF to agent that promotes call survival or invasion [79]. For instance, either mutations or dysregulation in these receptors might cause aberrant signaling that promotes cancer progression [80].

In addition, tumor microenvironment factors, such as the presence of immune cells, growth factors, and extracellular matrix components can modify PEDF’s behavior, which may enhance tumor cell survival or proliferation. This dual nature highlights the importance of understanding the context-specific effects of PEDF in cancer therapy [81].

## 3. PEDF in Selected GI Malignancies

Previous studies have highlighted the crucial role of PEDF in various stages of carcinogenesis, such as cancer angiogenesis, its growth, and the formation of distant metastases. Consequently, this protein has become a focal point of interest for many scientists [82]. Nevertheless, the precise mechanisms that are responsible for the development of cancer remain quite controversial. However, PEDF’s inhibitory effect on certain cancers has drawn attention as a potential target in anticancer therapy [83,84]. Recent research has strongly indicated that changes in the endogenous expression of PEDF are associated with the malignant progression of many cancers, alongside the well-known anticancer activity of exogenously administered PEDF [73]. Immunohistochemical analysis of PEDF expression in both various human tumor samples and healthy control tissues showed that increased PEDF expression is associated with better patient prognosis, whereas reduced levels indicate a worse prognosis [82,85,86].

PEDF expression in PC was investigated by Uehara et al. [87]. Their study aimed to investigate whether PEDF expression in tumor tissue could lead to low microvessel density (MVD) and to investigate its association with the presence of liver metastases and prognosis. The authors assessed PEDF expression using immunohistochemistry (IHC) in relation to MVD, clinicopathological tumor features, and the survival of PDAC patients. PEDF expression was found to be significantly associated with MVD. However, according to clinicopathological features, no significant correlations have been observed between expression of tested protein and depth of tumor invasion, lymphatic and venous invasion, or histopathological grading. Thus, it was shown that low PEDF expression was associated with an increased risk of liver metastases, as well as shorter patient survival. These results are consistent with other reports in which the extent of angiogenesis within the tumor was considered a critical factor in determining metastatic potential. Given the strong correlation between vascular endothelial growth factor (VEGF) expression and MVD in PDAC and its association with liver metastasis, these findings may suggest that the balance between PEDF and VEGF plays a crucial role in regulating both MVD and the development of liver metastases. Another research evaluated whether PEDF overexpression by gene transfer could block tumor angiogenesis and reduce tumor growth. In the evaluated model, significantly lower MVD density was observed in PEDF-treated tumors compared to those in the control group. A significant relationship between PEDF expression and MVD, stage, presence of liver metastases, and overall patient prognosis was demonstrated [88]. Similar results were obtained by Samkhadze et al. [89], whose results show a related correlation between higher PEDF expression and longer patient survival. Furthermore, silencing PEDF in PC cells resulted in reduced inhibition of endothelial cell growth in vitro, implicating PEDF in nerve density and morphology. Notably, there was a significant reduction in nerve density, as evidenced by a decrease in their numbers within the assessed area compared to normal pancreas tissue. Immunohistochemical studies demonstrated stronger PEDF expression in the activated pancreatic stroma, where lesions resembling chronic pancreatitis lead to swelling around the tumor. Furthermore, the authors suggested that the focal increase in PEDF expression in PDAC contributes to tissue hypoxia by inhibiting angiogenesis [81]. However, in addition to its well-known antiangiogenic effects, PEDF has also been found to exert fibrogenic effects by activating PSCs and promoting the production of extracellular matrix (ECM) proteins. It is highly likely that the combined effects of both fibrosis and peribronchiolar hypoxia may outweigh the neuroprotective effects of PEDF on small nerve fibers. Given that PEDF is a potent neurotrophic and neuroproliferative factor, its high focal expression in some PDAC patients strongly correlates with intrapancreatic neuropathy [89].

Pancreatic ductal adenoma (PDAC) is characterized by marked tumor-related inflammation, including macrophage infiltration and epithelial mutations in the KRAS gene [90]. However, reports on the interactions between tumor epithelium and macrophages at the early stage of carcinogenesis remain questionable. The above issue was addressed by Bishehsari et al. [91], who, using organoid cultures, showed that the most common KRAS mutations in human PDAC lead to a strict neoplastic transformation of the epithelium by changing macrophages to a strictly pro-tumor phenotype. This was assessed by the increase in macrophage gene expression, as well as their ability to initiate colony formation. KRAS mutations, as highlighted in the manuscript, activate downstream MAPK and PI3K/Akt signaling pathways that promote cell survival and proliferation. PEDF may antagonize these oncogenic signals, further inhibiting tumor growth [91]. Additionally, the authors described one of the pathways that may potentially participate in the influence of tumor-associated macrophages on the tumor epithelium. It was discovered that the expression of PEDF, which is a PDA tumor suppressor, decreases in the transforming epithelium not strictly due to the KRAS mutation, but under the influence of macrophages. EDF/EGFR signaling has been found to be a common pathway by which macrophages influence PEDF levels in pancreatic cancer epithelial cells. So far, no direct relationship between the EGF/EGFR axis and PEDF has been reported; however, it may be related to the induction of MMPs. These findings suggest that the reduction in PEDF levels in the epithelium may occur through the microenvironment, which may additionally facilitate PDAC progression through known mechanisms such as antiangiogenic or proapoptotic effects. In addition, in pancreatic neoplasia, epithelial–macrophage crosstalk, as discussed by Bishehsari et al. [91], is essential in the tumor microenvironment. PEDF has been shown to reduce inflammation by inhibiting NF-κB signaling and reducing the secretion of pro-inflammatory cytokines such as IL-6 and TNF-α, which are key players in tumor-associated inflammation. Further studies could help determine whether PEDF could be a potential target to compensate for the pro-tumorigenic effect of macrophages on the epithelium. Another theory regarding macrophage activity in pancreatic cancer was presented by Principe AR et al. [92], who showed that, concomitant with the lack of PEDF, the inflammatory profile of the tumor was characterized by macrophage infiltration. This suggests that PEDF inhibits macrophage activity and recruitment. Although these results may indicate negative regulation of inflammation by PEDF, it is suggested that PEDF has a pro-inflammatory effect. These observations contrast with studies of PEDF in prostate cancer, in which the anti-tumor properties of PEDF were associated with greater macrophage recruitment, which may be due to the tissue-specific effects of PEDF [92]. Consequently, the ability to recruit macrophages with a pro-tumor or anti-tumor phenotype is dependent on the tumor’s chronology. Finally, macrophages suppress PEDF, leading to angiogenesis and tumor progression, which is consistent with the conclusions of Bishehsari et al. [91]. The severity of inflammation and fibrosis in the pancreas ultimately leading to tumorigenesis through loss of PEDF was investigated by Principe AR et al. [92]. Initially, the authors assessed the relationship between PEDF and inflammation associated with pancreatic cancer using IHC methods. Significantly reduced PEDF expression was demonstrated in malignant sections, indicating that PEDF correlates inversely with inflammation [92]. To verify whether loss of PEDF could contribute to the increased risk of pancreatitis in vivo, the effect of caerulein-induced acute pancreatitis was assessed in both control and PEDF-null (−/−) mice. PEDF (−/−) mice were shown to develop more severe pancreatitis compared to a control group of wild-type mice. These data indicate that loss of PEDF increases the risk of pancreatitis. Regarding the contribution of PEDF to pancreatic fibrosis, the authors observed in vitro and in vivo that tumor sections with complete loss of PEDF had significantly higher fibrosis scores than those with high PEDF expression. Moreover, TGFβ1 expression was increased in PEDF samples and was statistically significantly lower in PEDF-samples [92], which may indicate that PEDF is involved in the inhibition of fibrosis in pancreatic cancer via TGFβ1. These results contradict those presented by Samkhadze et al. [89].

The clinical significance of PEDF as a potential indicator of PC progression has been investigated by Edderkaoui M et al. [93]. First, the authors measured the amount of PEDF mRNA in human and mouse tumor tissue. A statistically significantly higher level of mRNA was found in the tissue of PC mice than in the control group. Then, the level of PEDF in the blood of patients with PDAC, pancreatitis and healthy people was also assessed, and showed a significantly higher PEDF concentration in PDAC compared to the control group and people with pancreatitis [93]. These results are contrary to those of studies conducted by Uehara H et al. [87], who showed a correlation of low PEDF concentration with tumor progression, which outweighs the anticancer effect of PEDF. The specific mechanism by which increased PEDF expression is responsible for PC progression is not fully known. However, a similar relationship related to PEDF expression in GI tumors was demonstrated in ESCC in the study by Tang DR et al. [94]. The authors demonstrated PEDF overexpression in ESCC tissues and cells. Moreover, it was observed that PEDF reduction using shRNA significantly inhibited both the proliferation and invasion of ESCC in vitro and in vivo [94]. Grippo PJ et al. have evaluated PEDF deficiency and Kras mutations as a potential factor for inducing invasive PC and adipose-rich tumors in mice [95]. The authors showed that EL-Kras/PEDF-deficient mice had developed invasive PDAC, which was associated with intensified MMP-2 and MMP-9 expression as well as with increased intrapancreatic fat. This was accompanied by adipocyte hypertrophy and intrapancreatic adipocyte infiltration. Additionally, the authors examined the concentrations of both PEDF and VEGF in the serum of PC patients compared to the control group. It was shown that cancer patients had statistically lower PEDF concentrations and higher VEGF concentrations compared to healthy people. Moreover, the VEGF/PEDF ratio was three times higher in cancer patients than in people without malignant lesions, which may suggest the potential use of PEDF as a biomarker for PDAC in a combined analysis with VEGF [95].

The expression of PEDF and its interaction with two putative receptors in HCC have been evaluated by Akiba J et al. [86]. The study showed that high PEDF expression was associated with poor HCC prognosis. The authors revealed that high LR expression in HCC was associated with an aggressive clinicopathological variant of HCC, while high PEDF expression was associated with a less malignant phenotype. Both PEDF and its receptors LR and ATGL have been shown to be significant in HCC and liver tissue. Consequently, the above parameters can be used as potential biomarkers, especially with regard to clinical prognosis [96]. Moreover, they may also constitute potential therapeutic targets for both cancer and other chronic liver diseases. Similar results were obtained by Hou J et al. [52]. The authors showed that PEDF overexpression both in vivo and in vitro can significantly increase the migration as well as metastasis of HCC cells by interacting with the LR. Moreover, the combined expression of PEDF, LR and N-cadherin was shown to be more sensitive than expression of either one alone when it came to overall survival (OS) [52]. Therefore, these results suggest that the generated PEDF-LR complex may be a potential biomarker of HCC prognosis, and its use may also be used as a therapeutic target. Li C et al. studied the intra- and extracellular functions of PEDF in HCC [97]. The authors showed that PEDF is not related to the prognosis of patients, although strong expression of this protein was observed in the tissues of such tumor. Nevertheless, they also elucidated findings indicating that intracellular PEDF resulted in the accumulation of free fatty acids (FFAs). Consequently, it promoted the growth of HCC cells. On the other hand, PEDF secretion and its function as an antiangiogenetic agent were described, and it was found to have the ability to inhibit tumor angiogenesis in HCC [97]. The antiangiogenic effect of PEDF in HCC was also confirmed by Wang X et al. [98]. The authors focused on the p18 peptide, which is a specific fragment of PEDF. With the rapid growth of solid tumors, the hypoxic environment inside tumor tissues stimulates the expression of matrix metalloproteinases and pro-angiogenic cytokines such as MMP-2 and MMP-9. These mentioned metalloproteinases are present in HUVEC and play a role in tissue remodeling during carcinogenesis, cancer metastasis, and angiogenesis. Moreover, research conducted by Wang X et al. [98] showed that additional VEGF leads to an increase in the secretion of both MMP-2 and MMP-9 in HUVEC cells. Based on the results obtained, it was demonstrated that the P18 peptide is capable of controlling angiogenesis by blocking the VEGF/VEGFR2 axis. Simultaneously, the P18 peptide inhibits cancer cell viability by impeding cell migration rather than inducing apoptosis in HCC [98]. Li-Ju L et al. studied the relationship of c-FLIP and PEDF in HCC [99]. In this study, PEDF was shown to play a critical role in promoting apoptosis in HCC cells, especially by enhancing the apoptotic effects of ciglitazone, a synthetic ligand for PPARγ (Peroxisome Proliferator-Activated Receptor Gamma). It has been shown that downregulation of c-FLIP dramatically renders HCC cells sensitive to death receptor apoptosis. Moreover, PEDF downregulates the expression of both c-FLIP mRNA and protein through p38 kinase signaling; therefore, in addition to its antiangiogenic nature towards endothelial cells, it may also influence the survival of HCC cells. In the context of HCC, PEDF acts as a tumor suppressor by promoting apoptosis, particularly through the inhibition of c-FLIP, a key regulator of the extrinsic apoptotic pathway. By reducing c-FLIP levels, PEDF facilitates the activation of death receptors like Fas, leading to caspase-8 and caspase-3 activation and subsequent cell death. When combined with ciglitazone, PEDF amplifies the apoptotic effect, offering a potential therapeutic approach for treating HCC [99]. Thus, it leads to the conclusion that PEDF expression is associated with HCC progression, and the search for a direct effect of PEDF on HCC is crucial in establishing new therapeutic strategies [99].

The effects of PEDF on various GI cancers were presented in Table 1.

## 4. PEDF as a Potential Therapeutic Target for Cancer

While recent epidemiological findings may suggest a decline in the incidence of certain cancers, they continue to be among the key causes of mortality worldwide. Therefore, a significant challenge is to enhance current therapeutic strategies especially those aimed at inhibiting tumor growth, the survival of cancer cells, and metastasis. PEDF, a non-inhibitory serpin, is now widely expressed throughout the body. Although initially identified as a factor involved in neuronal differentiation, more recent attention has been directed toward its antiangiogenic activity, which holds promise for anticancer therapy [100,101]. The strongest support for the role of PEDF in anticancer therapy comes from findings that this molecule exhibits antiangiogenic and anti-metastatic activity. PEDF, as a natural inhibitor of angiogenesis, blocks the formation of new blood vessels by starving tumors of nutrients and oxygen via VEGF inhibition. PEDF can also trigger apoptosis in cancer cells, contributing to the reduction in tumor mass through mechanisms such as activation of JNK and p38 MAPK signaling pathways, both of which are involved in stress responses and promote apoptosis or inhibition of the AKT pathway, which normally promotes cancer cell survival and proliferation. By suppressing AKT, PEDF makes cancer cells more prone to apoptosis [102]. PEDF also plays a role in reducing both chronic inflammation and oxidative stress, which are often associated with tumor progression and metastasis. By exhibiting anti-inflammatory properties, it may help create a less favorable environment for cancer growth [103]. Furthermore, PEDF’s antioxidant activity reduces oxidative stress in cancer cells, potentially compromising their survival, since cancer cells often rely on higher levels of reactive oxygen species for proliferation [103]. Another area in which PEDF has shown potential in anticancer therapy is the suppression of cancer stem cells (CSCs) growth and differentiation, which makes tumors more sensitive to standard therapies and reduces the likelihood of disease recurrence [104]. One of the biggest advantages of PEDF is its low toxicity to normal tissues, which makes it a safer option compared to many conventional anticancer drugs that have severe side effects [105]. PEDF targets cancer-specific pathways like abnormal angiogenesis and cancer cell survival mechanisms, sparing healthy cells from damage. The real utility of PEDF lies in its synergy with conventional cancer therapies. It has been suggested that PEDF could be used in conjunction with other cancer treatments, including chemotherapy, radiotherapy, and targeted therapies, to improve therapeutic outcomes. This approach would enhance the effects of chemotherapy and radiotherapy by increasing the sensitivity of cancer cells to these treatments [106]. Furthermore, it has great potential to reduce side effects by protecting normal cells from oxidative damage and promoting their survival, which could improve the overall quality of life of patients undergoing treatment. Furthermore, researchers have demonstrated that exogenous administration of PEDF to boost declining levels of this protein within the tumors during tumor progression leads to the inhibition of tumor growth and prolonged survival in various animal models [107,108]. Given PEDF’s involvement in various biological processes, such as angiogenesis and metastasis inhibition, its in vivo anticancer efficacy was assessed in a metastatic mouse lung model inoculated with CRC cancer [83,109]. Following PEDF treatment, the condition of the lungs was examined, and no significant differences were observed compared to normal lungs [110]. In addition, treatment with PEDF led to a reduced number of metastatic nodules. Furthermore, protocols involving combination therapy with PEDF and differentiation inducers such as IL-6 have also been investigated [111]. In the context of a therapeutic agent, two primary methodologies for assessing PEDF are considered. One approach involves utilizing this protein in gene therapy. In this model, PEDF is administered in a form that is expressed in a viral vector and there is evidence that it is effective [112,113]. The second model involves administering unmodified PEDF directly to the tumors, resulting in tumor regression due to PEDF’s inhibitory effects on both the tumor itself and its vasculature. It is suggested that these models could enhance both the pharmacokinetics and pharmacodynamics of administered drugs. However, a fundamental constraint in all approaches is the need to constantly supply the PEDF gene to maintain the expected expression in tumors. Additionally, since this protein is also expressed in normal organs, such actions may impact the functions of healthy organs [60,114].

Currently, researchers are exploring a novel approach using platinum-based chemotherapeutic drugs to increase PEDF expression [115]. The use of both conventional cis-platin and its derivatives is associated with their role as chemotherapy agents that primarily target DNA. The mechanism of action involves binding to the nuclear DNA of cancer cells and causing distortion of DNA conformation. As a consequence, a cascade of cellular mechanisms is triggered, which, if left unrepaired, leads to the inhibition of transcription and subsequent induction of apoptosis of cancer cells. One promising platinum complex is (1R,2R-diaminocyclohexane)(dihydropyrophosphato)platinum(II), abbreviated as RRD2, which falls into a category of potent cytostatics known as phosphaplatins [116].

In conclusion, PEDF has broad utility in anticancer therapy due to its ability to disrupt multiple pathways that are critical to tumor growth, spread, and survival, making it a promising candidate for future cancer treatments.

## 5. Conclusions

The recent significant progress in understanding the pathomechanisms of cancer formation and progression in humans has led to improvements in therapeutic procedures. Nevertheless, the fight against cancer continues, and for many cancers, new alternatives are still being sought to provide us with a more detailed understanding of cancer’s biology and to help us discover optional biomarkers for these diseases. The anticancer effects of PEDF have only been recently investigated, and more time and work are needed to elucidate additional details about the signaling cascades and mechanisms of this protein and its biological role in GI cancers. The potential of PEDF lies in its combined use alongside other cancer therapies, such as chemotherapy or radiation therapy, to enhance their effectiveness. It may sensitize cancer cells to the effects of these treatments, making them more vulnerable to destruction. Understanding the mechanisms by which PEDF exerts its effects on cancer cells could lead to the development of targeted therapies that mimic its actions or enhance its natural production within the body. However, it is extremely important to note that while PEDF holds promise as a therapeutic agent in cancer, more research, including clinical trials, is needed to fully evaluate its safety and efficacy in different types of cancer. The specific utility of PEDF may vary depending on the cancer type and the individual patient, and it is likely to be part of a broader treatment approach rather than a standalone therapy. However, potential challenges to the utility of PEDF as a part of the anticancer therapy include both delivery mechanisms and potential cancer cell resistance. Effective delivery of PEDF or PEDF-based therapies to the tumor site remains challenging, and developing methods to increase its bioavailability is an ongoing area of research. On the other hand, as with many current therapies, there is a risk that tumors may develop resistance to PEDF, requiring strategies to maintain its efficacy over time.

## Figures and Tables

**Table 1:** The effect of PEDF on PC and HCC.

Cancer	PEDF Effect	Method of Analysis	Potential Molecular Signaling Involved in Effect	References
Pancreatic cancer (PC)	PEDF as indicator of PC progression	IHC	ND	[87]
	PEDF as indicator of PC progression	RT-PCR, ELISA	ND	[93]
	Fibrogenic	WB	PSCs	[89]
	Anti-inflammatory	IHC	JNK	[92]
	Anti-fibrotic	IHC	TGFβ	[92]
Hepatocellular cancer (HCC)	HCC apoptosis and progression	WB	Caspase-3, p38	[99]
	HCC less aggressive phenotype	IHC	LR	[96]
	HCC cells migration and metastases	IHC	LR	[52]
	HCC growth via FFA	Colorimetric	AMPK	[97]
	Tumor angiogenesis inhibition	IHC, IF	VEGFR-2	[98]

Abbreviations: AMPK—5′AMP-activated protein kinase; ELISA—enzyme-linked immunosorbent assay, FFA—free fatty acids, HCC—hepatocellular carcinoma; IF—immunofluorescence, IHC—immunohistochemistry, JNK—c-Jun N-terminal kinase, LR—laminin receptor, ND—no data available, PC—pancreatic cancer; PSC—primary scrambling code, RT-PCR—real time polymerase chain reaction, TGF—tumor growth factor, WB—Western Blot, VEGFR-2—vascular endothelial growth factor receptor-2.
